# Retrospective Diagnosis of Recurrent Takotsubo Syndrome Episodes in a 40-Year-Old Woman

**DOI:** 10.3390/diagnostics16132083

**Published:** 2026-07-03

**Authors:** Malgorzata Zalewska-Adamiec, Hanna Bachorzewska-Gajewska, Slawomir Dobrzycki

**Affiliations:** 1 Department of Invasive Cardiology, Internal Medicine with CICU and Laboratory of Hemodynamics, Medical University of Bialystok, 15-089 Bialystok, Poland; 2 Department of Invasive Cardiology, Internal Medicine with CICU and Laboratory of Hemodynamics, University Hospital in Bialystok, 15-089 Bialystok, Poland

**Keywords:** Takotsubo syndrome, reccurent Takotsubo, InterTAK score, coronary angiography, left ventriculography

## Abstract

Recurrence of Takotsubo syndrome (TS) is the most significant challenge in the long-term follow-up of patients with TS. We present the case of a 40-year-old woman with a history of two acute coronary syndromes and myocarditis who was hospitalized in our clinic due to Takotsubo syndrome. Left ventriculography demonstrated the rare mid-ventricular variant of Takotsubo syndrome. Based on a carefully obtained medical history, it was established that all previous coronary events, as well as the current TS episode, had been preceded by psychological stress. Following an additional review of the patient’s medical records and application of the InterTAK score, we concluded that the previous incidents may have been episodes of TS and that the patient had now experienced her fourth episode of Takotsubo syndrome.

**Figure 1:**
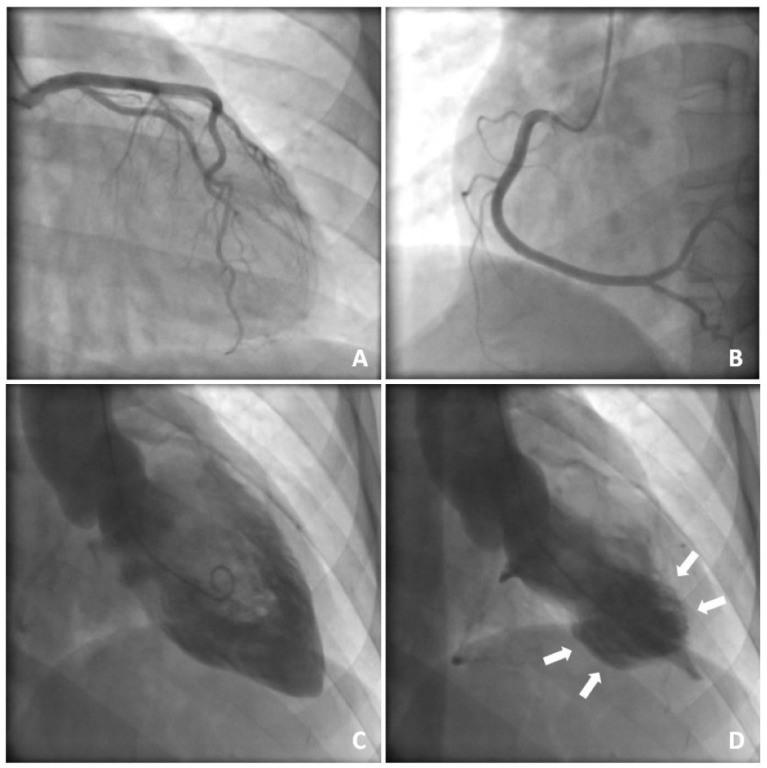
Takotsubo syndrome (TS) is a transient disorder of regional left ventricular contractility with a clinical presentation resembling acute coronary syndromes. Serious complications may occur during the acute phase of the disease, whereas in long-term follow-up, the main concern is TS recurrence [1,2]. A 40-year-old woman with hypothyroidism and mixed hyperlipidemia, presenting with several hours of resting retrosternal chest pain preceded by a stressful event, was admitted to our clinic with suspected inferior ST-segment elevation myocardial infarction (STEMI). Ten years earlier and one year earlier, she had experienced two acute coronary syndromes, and one month before admission she had been diagnosed with myocarditis. At admission, the patient was in stable condition and reported no chest pain. Blood pressure was 137/84 mmHg and BMI was 23.66 kg/m^2^. Electrocardiography showed sinus rhythm at 60 bpm, intermediate axis, first-degree atrioventricular block, ST-segment elevation in leads II, III, and aVF, and a corrected QT interval (QTc) of 478 ms. Laboratory tests revealed elevated troponin levels of 6541 ng/L (normal < 15.60 ng/L) and NT-proBNP of 578.8 pg/mL (normal < 125 pg/mL). Echocardiography demonstrated normal cardiac chamber dimensions, preserved valvular function, and regional left ventricular wall motion abnormalities, including akinesia of the mid-inferior and mid-inferolateral segments and hypokinesia of the mid-interventricular septal, lateral, and anterior segments, with a left ventricular ejection fraction (LVEF) of 54%. Coronary angiography revealed normal coronary arteries (**A**,**B**), whereas left ventriculography demonstrated wall motion abnormalities involving the mid-segments of the left ventricular walls (**C**,**D**, white arrows), consistent with the mid-ventricular variant of Takotsubo syndrome, with an LVEF of 52%. Following a detailed review of the patient’s medical records and additional history regarding her previous coronary events and myocarditis, we found that all episodes had been preceded by psychological stress. Using the InterTAK Diagnostic Score, we concluded that these three previous events may have been episodes of Takotsubo syndrome (apical variants), and that the current episode represented another recurrence of TS. The hospital course was uneventful. Treatment included aspirin, ramipril, and bisoprolol, but the beta-blocker was discontinued due to bradycardia and first-degree atrioventricular block. The patient was discharged after five days. At discharge, treatment with ramipril 2.5 mg/day, acetylsalicylic acid 75 mg/day (for one month), and levothyroxine was prescribed. Follow-up echocardiography performed three weeks later demonstrated complete recovery of left ventricular systolic function. Recurrence of Takotsubo syndrome is the serious challenge in long-term follow-up. It affects approximately 1–10% of patients with TS. According to the most recent 2024 consensus statement, recurrence of Takotsubo syndrome is underdiagnosed and insufficiently described in the literature. Some cases diagnosed as a first episode of TS may in fact represent recurrent TS, because previous episodes had been misdiagnosed as acute coronary syndromes. Therefore, the true recurrence rate of Takotsubo syndrome is likely underestimated. Factors associated with an increased risk of TS recurrence include physical and psychological stress, severe left ventricular dysfunction during the initial episode, postmenopausal age, malignancy, arterial hypertension, and neurological and psychiatric disorders. Recurrence may occur from one month to several years after the initial episode and may happen repeatedly. Recurrent TS episodes are most often triggered by psychological stress, and the pattern of regional wall motion abnormalities may vary in the same patient [3,4,5,6]. Preventive strategies remain limited, and the results of completed and ongoing studies on the prevention of TS recurrence remain inconclusive. Psychological and/or psychiatric care is recommended for patients following an episode of Takotsubo syndrome. Pharmacological treatment includes angiotensin-converting enzyme inhibitors/angiotensin receptor blockers and beta-blockers. Recent reports have also demonstrated a beneficial effect of SGLT2 inhibitors, which, by reducing myocardial edema and improving left ventricular systolic function, may improve long-term survival in patients with TS [7]. Using this case as an example, we demonstrate that a thorough analysis of previous coronary events in patients with Takotsubo syndrome, together with the application of the InterTAK Diagnostic Score, can facilitate reassessment of prior diagnoses and identification of recurrent TS.

## Data Availability

Original data supporting the reported results are available by contacting the authors.
